# Elevated IL-35 level and iTr35 subset increase the bacterial burden and lung lesions in *Mycobacterium tuberculosis*-infected mice

**DOI:** 10.1515/biol-2022-0025

**Published:** 2022-03-31

**Authors:** Fangliu Yu, Xinying Zhu, Qingdeng Li, Wenqin Xu, Yunxing Gao, Yufeng Wen, Qiong Zhang, Jun Dou

**Affiliations:** Department of Medical Microbiology and Immunology, School of Preclinical Medicine, Wannan Medical College, Wuhu 241001, PR China; School of Public Health, Wannan Medical College, Wuhu 241001, PR China; Center of Disease Control and Prevention, Wuhu, Anhui, 241001, PR China; Departments of Pathogenic Biology and Immunology, Medical School, Southeast University, 87 Ding Jiaqiao Rd., Nanjing 210009, PR China

**Keywords:** *Mycobacterium tuberculosis*, H37Ra, IL-35, IL-35-secreting regulatory T cells, immune suppression

## Abstract

This study aimed to investigate the relationship between interleukin (IL)-35 level and IL-35-producing regulatory T cells (iTr35 subset) in *Mycobacterium tuberculosis* (Mtb)-infected mice. After the mice were injected with Mtb strain H37R via tail vein, the bacterial burden, lung lesions, and the impact of immune suppression on the infected mice were respectively assessed. The results, when compared with the control mice, showed that the mRNA expression levels of the p35 and Epstein-Barr virus-induced gene 3 of IL-35 were significantly increased in the Mtb-infected mouse spleen at 4 or 8 weeks post-infection and their protein expression levels were concurrently increased in the lungs of the mice, especially in 8 week infected mice. In addition, the levels of serum IL-35 and the iTr35 subset in the spleen of mice were also increased in 4 or 8 weeks post-infection compared with the control mice. Importantly, the high bacterial burden and lung lesions and the low mouse weight were found at 8 week post-infection. Therefore, the mice infected with Mtb resulted in elevating IL-35 level and iTr35 subset and increasing bacterial burden and lung lesions. The findings from the study suggest IL-35 and iTr35 cells may exert an immune suppression role in chronic Mtb-infected mice.

## 1 Introduction

*Mycobacterium tuberculosis* (Mtb) infection causes human tuberculosis (TB), and is still a major cause of morbidity and mortality worldwide. More than two billion people are infected with TB all over the world, and up to one-third of people in a chronic TB-infectious state are at risk of developing reactiveness TB [1,2]. The TB vaccine developed by *Mycobacterium bovis* bacillus calmette guerin (BCG) is permitted for use in humans; however, some proteins, for example, 6 kDa early secretory antigen target, were lost in BCG [3], which cause BCG’s immune immunogenicity weakness, and does not confer protection against the establishment or reactivation of a latent TB infection. Therefore, new vaccine candidates are needed to address this need [4] specifically. Moreover, BCG shares many of the immune evasion proteins utilized by Mtb, but the role of these proteins in immune cell responses elicited by BCG is poorly understood [5].

Published studies suggest that the Mtb infection of naïve individuals for weeks was not restricted by IFNγ-producing immune cells, which finally pile up and set up the chronic infection of Mtb [6,7]. In active TB patients, the serum interleukin (IL)-35 level was increased, which is the newest member IL-12 family of cytokines. IL-35 consists of two separate dimeric subunits and has a probative inhibitory effect on the immune system by the development of IL-35-secreting regulatory T cells (iTr35), which increase regulatory T cells (Tregs) formation [8,9,10]. Epstein-Barr virus-induced gene 3 (EBI3), a secreted form, is a subunit of immunoregulatory cytokine IL-35, and its effects have been assessed and explored via extracellular EBI3 [11]. Currently, we do not know the IL-35 level, iTr35 cell subset, and expressions of EBI3 and p35 (another subunit of IL-35) in the chronic Mtb-infected hosts.

In this study, we tested the serum IL-35 level, the number of iTr35 cell subset, and both p35 and EBI3 expression levels in 4 or 8 weeks Mtb-infected mice via tail vein injection of Mtb to evaluate whether these immunological molecules and iTr35 cells associated with the immune suppression would influence the bacterial burden and lung lesions in chronic Mtb-infected mice. Here, we report that the lesions of lungs, the protein expression levels of both EBI3 and p35, as well as the number of iTr35 cell subset were respectively increased in C57BL/6 male mice injected with Mtb H37Ra at 8 week post-infection and enhanced serum IL-35 level was observed in mice at 4 or 8 weeks post-infection. The decrease of mouse weight, an increase of bacterial burden, and lung lesions were also observed in the established mouse model, which was the Mtb-infected mice at 8 weeks post-infection. Our data indicate that an increase in bacterial load and lung lesions is related to augmentation of levels of IL-35, EBI3, P35, and iTr35 cell subset count in the chronic Mtb-infected mice where the immune suppression may play an important role.

## 2 Materials and methods

### 2.1 H37Ra

The attenuated H37Ra strain of Mtb was provided by the Jiangsu Province Centers for Disease Control and Prevention, PR China. The attenuated Mtb H37Ra was cultured in a Sauton medium that includes 0.5% sodium pyruvate and 0.5% glucose. In the study, the viable attenuated H37Ra strain of Mtb was used as the challenge study.

### 2.2 Mice

C57BL/6 male mice (age between 5 and 6 weeks) were supplied as specific-pathogen-free by the Animal Center of Qinglong mountain, Jiangning Zone, Nanjing of China, license number SCXK (Jiangsu) 2017-0001. Mice were raised under an SPF level animal facility at the Experimental Animal Center, Wannan Medical School, Wuhu, PR China.


**Ethical approval:** The research related to animal use has been complied with all the relevant national regulations and institutional policies for the care and use of animals. Animal experiments were performed in compliance with the guidelines of the Animal Research Ethics Board in Southeast University, PR China.

### 2.3 Mouse TB-infected model

Twenty male C57BL/6 mice were infected by tail vein injection with 1 × 10^5^ colony-forming units (CFU) of attenuated H37Ra strain of Mtb in a volume of 200 μL, in which 10 mice were executed at 4 weeks, and the remaining mice were monitored over an 8 week period. As a control, 10 mice were infected by tail vein injection with 200 μL phosphate buffered saline (PBS) [3,12]. The experiment was repeated twice. The H37Ra strain of Mtb-infected mouse model was successfully established according to identifying the bacteria morphology in each mouse lungs.

### 2.4 CFU, body mass index (BMI), and spleen coefficient

The CFUs in the left lung were assessed at 4 and 8 weeks Mtb-infected mice by serial dilutions of left lung homogenates. The lung homogenates suspensions, including bacteria after lysis with saponin 0.1% in distilled water, was enumerated by plating 10-fold dilutions, prepared in distilled water, on a Sauton medium enriched with 0.5% sodium pyruvate, 0.5% glucose, 50 μg/mL carbenicillin, and 20 μg/mL trimethoprim. The CFU count was determined 21 days after incubation at 37°C in humidified air. CFUs were counted visually [13,14]. The BMI and spleen coefficient in infected mice were detected following the formula [15]:

\text{BMI}=\text{Mass}\hspace{.5em}\text{post}\hspace{.5em}\text{infection/Mass}\hspace{.5em}\text{before}\hspace{.5em}\text{infection}\times 100 \% ,]

\text{Spleen}\hspace{.25em}\text{coefficient}=\text{Spleen mass/Mouse}\hspace{.25em}\text{mass}\times 100 \% .]

### 2.5 Histology and morphometry

For morphometry, 5 μm sections were stained with hematoxylineosin (HE) for routine evaluation in a blinded manner with Alizarin red to detect calcified lesions by two investigators. To evaluate the histopathological parameters including peribronchiolitis, perivasculitis, alveolitis, and granuloma formation, we used each semiquantitative scored as absent, minimal, slight, moderate, marked, or strong, numbered 0, 1, 2, 3, 4, and 5, respectively. The frequency and severity of the lesions in this score were incorporated [16,17].

### 2.6 Quantitative real-time polymerase chain reaction (qRT-PCR)

qRT-PCR analysis was carried out on an ABI step one plus real-time system (Applied Biosystems). We used a Qiagen RNeasy Kit (Qiagen, Valencia, CA) to isolate total cellular RNA from each sample. One microgram of total RNA from each sample was subjected to cDNA synthesis using the Superscript III reverse transcriptase (Invitrogen). cDNAs were amplified by PCR with primers as following: EBI3 (sense, 5′-CATTGCCACTTACAGGCTCG-3′; antisense, 5′-GGATGT ACGATTTACAGTGACGT-3′); p35 (sense, 5′-CAATCACGCTACCTCCTCTTT T-3′; antisense, 5′-CTTTGTAATAAGGACGTGACGAC-3′); β-actin (sense, 5′-GGCTGT ATTCCCCTCCATCG-3′; antisense, 5′-TGTACCGTAACAATGGTTGACC-3′). The ratio of each interest gene to β-actin for each sample was noted as mRNA expressive level of the interest genes. SYBR green quantitative PCR amplifications were performed in the Step one plus Detection System (Applied Biosystems). The comparative Ct (ΔΔCt) method was adopted to definite the expression fold change [18,19]. All the primers were synthesized by Gene and Technology of China in Shanghai.

### 2.7 Western blot

The assay was performed as reported previously [20,21]. In brief, the lung suspensions were acquired from the 4 and 8 week infected mice and lyzed in the protein extraction buffer (Novagen, Madison, WI, USA) by following the manufacturer’s protocol. 12% sodium dodecyl sulfate-polyacrylamide gel electrophoresis was performed, and proteins (10 μg/lane) were transferred onto a polyvinylidene difluoride membrane blocked with 4% dry milk in tris-buffered saline with Tween-20 for 1 h at 20°C. The used antibodies included the APC anti-IL-27/35EBI3 (Rat anti-mouse, R&D, IC18341A) and PE anti-IL-12/35p35 (goat anti-mouse, R&D, IC2191P) for overnight at 4°C. The membrane was rinsed with an antibody wash solution for 5 min, in a total of three times before adding it to the goat anti-rat or the rabbit anti-goat fluorescence secondary antibody. Immunoreactive bands were detected by Odyssey scanning instrument (LI-COR Odyssey Imaging System, USA).

### 2.8 Cytokine IL-35 detection

Serum isolated from the 4 and 8 weeks infected mice were assayed for cytokine IL-35 using a commercially available double antibody sandwich enzyme-linked immunosorbent assay (ELISA) kit according to the manufacturer’s protocol (eBioscience Company, USA). Briefly, the detecting serum (1:1,000-fold dilution) was obtained in the infected mice, and IL-35 was captured by the specific primary monoclonal antibody and detected by a biotin-labeled secondary antibody. Plates were read at 450 nm using a microplate reader (Bio-Rad Labs, Hercules, CA). Samples and standards were run in triplicate, and the sensitivity of the assays was 0.1 units/mL for each cytokine [22,23].

### 2.9 Flow cytometer (FCM)

The analysis of forkhead box protein P3 (FOXP3)-expressing T cells was performed in accordance with the manufacturer’s instructions (eBioscience). In brief, the antibody was stained in mononuclear cells and rinsed with cold PBS. The cells were resuspended in the fix/perm buffer and incubated at 4˚C for 3 h, and washed twice in a permeabilization buffer. After that, anti-mouse/rat Foxp3-PE was added. The cells were incubated for 30 min and washed twice in PBS, resuspended in PBS, and analyzed in FCM. To CD4^+^- or CD25^+^-expressing T-cell analysis, the splenocyte suspensions were costained with the fluorescein isothiocyanate-conjugated anti-CD4 antibody, phycoerythrin (PE)-conjugated anti-p35-PE and EBI3-APC (eBioscience, CA, USA) antibodies for FCM detection in accordance with the manufacturer’s instructions [23,24].

### 2.10 Statistical analysis

Statistical comparisons of the results between the groups were performed using Student’s *t*-test or repeated-measures analysis of variance. *P* values less than 0.05 were considered to be statistically significant.

## 3 Results

### 3.1 Chronic Mtb-infected mice decrease BMI and increase spleen coefficient

**Figure 1:**
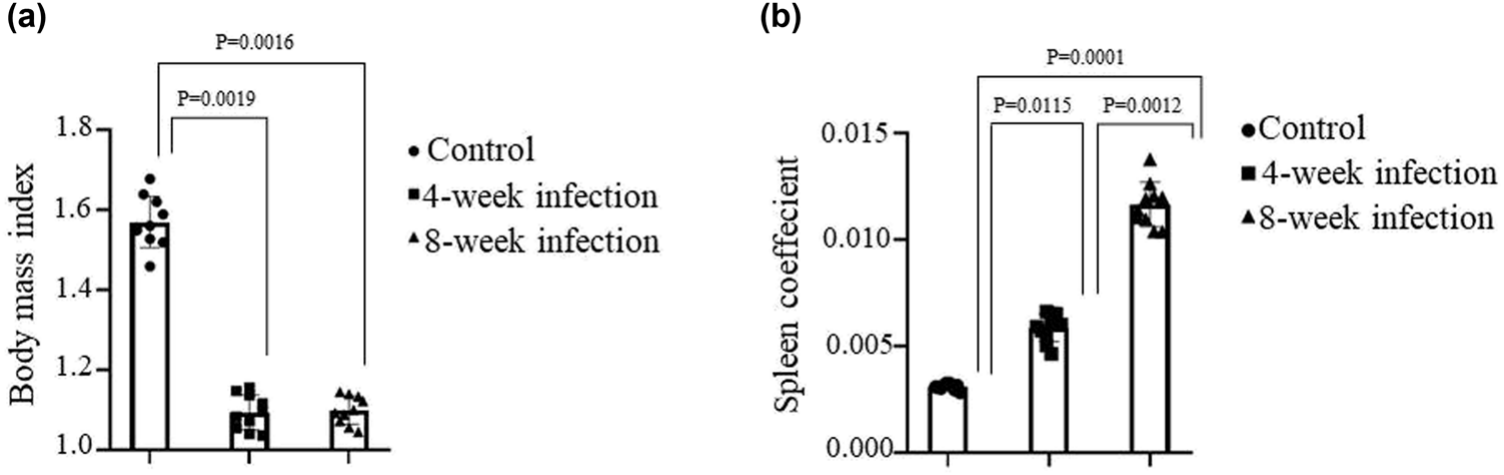
Detection of the BMI and spleen coefficient. Mice were infected by tail vein injection with 1 × 10^5^ CFUs of attenuated H37Ra strain of Mtb in a volume of 200 μL or PBS as control. Mice were executed at 4 or 8 week post-infection. (a) The BMI of mice in the different groups. (b) The spleen coefficient in the different groups. All the data represent mean ± SD (*n* = 10), referring to the differences as shown.

In order to establish the Mtb-infected mouse model, we first did the preexperiment in mice infected with the Mtb via tail vein injection with 1 × 10^5^ CFU of H37Ra strain. The lung’s viable bacteria were settled in whole-lung homogenates by serial dilutions and plating onto the nutrient medium in 24 h, and then the bacteria morphology was identified by acid-fast stain (data not shown). The results of acid-fast stain demonstrated that the Mtb-infected mouse model was established appropriately for further study of Mtb chronic infection. Figure 1a indicates the BMI of mice in various groups. It was found that the BMI was significantly descended in mice infected with Mtb for 4 and 8 weeks compared with infected mice, whereas the spleen coefficient was markedly enhanced in the 8 weeks infected mice (*p <* 0.0001) and in the 4 week infected mice (*p <* 0.0115) in contrast to the control mice (Figure 1b).

### 3.2 Pathological change and lung bacterial burden in chronic Mtb-infected mice

**Figure 2:**
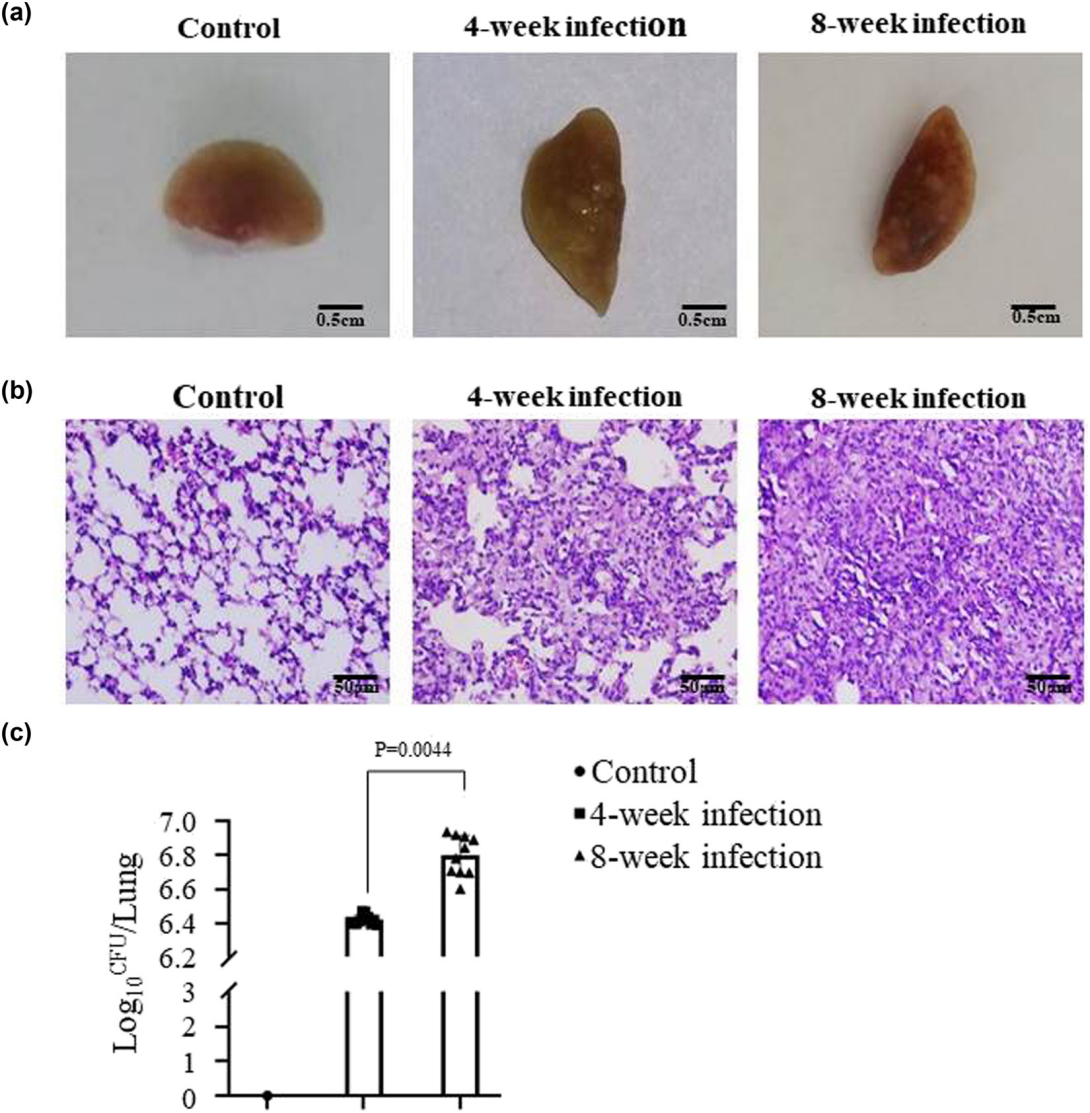
Mtb infection causes lung pathological change in mice. (a) The images of lung tissues fixed with 10% formalin at 4 or 8 weeks after the Mtb challenge the mice were killed. The control lung tissue (left), at 4 week Mtb-infected lung tissue (middle), and at 8 week Mtb-infected lung tissue (right). (b) The sections of the control alveolar architecture (left), the lung injury at 4 week post-infection (middle), and the lung injury at 8 week post-infection (right), which were stained with HE and observed under microscope (×200). (c) The statistical analysis of total numbers of CFUs in the different mouse groups. The histogram represents a set of data for 10 mice, referring to the differences as shown.

Figure 2a gives a representative clinical picture of different lung tissues fixed by formalin from the mice on 4 weeks without infection (left), 4 weeks (middle), and 8 weeks (right) post-infection of Mtb. It exhibits various tubercular nodules in lungs from the 4 or 8 weeks Mtb-infected mice, and no tubercular nodules were found in the lungs from the normal control mice. Figure 2b shows the pictures of histopathological change in mouse lungs. The left one exhibits the normal alveolar architecture without any infection and any pathological change, whereas the right one in Figure 2b shows that the lung injury was more serious, contained granulomas and TB lesions as well as less infiltrated inflammation cells and more epithelium-like change in mononuclear cells in the 8 weeks Mtb-infected mice than those in mice infected with Mtb for 4 weeks (middle one), which was a significant difference in the total numbers of CFU between the 8 weeks Mtb-infected mice and the 4 weeks Mtb-infected mice (6.655 ± 0.053 vs 6.431 ± 0.041, *p* < 0.0044) as is shown in Figure 2c.

### 3.3 The expression levels of EBI3, p35, and IL-35 in chronic Mtb-infected mice

**Figure 3:**
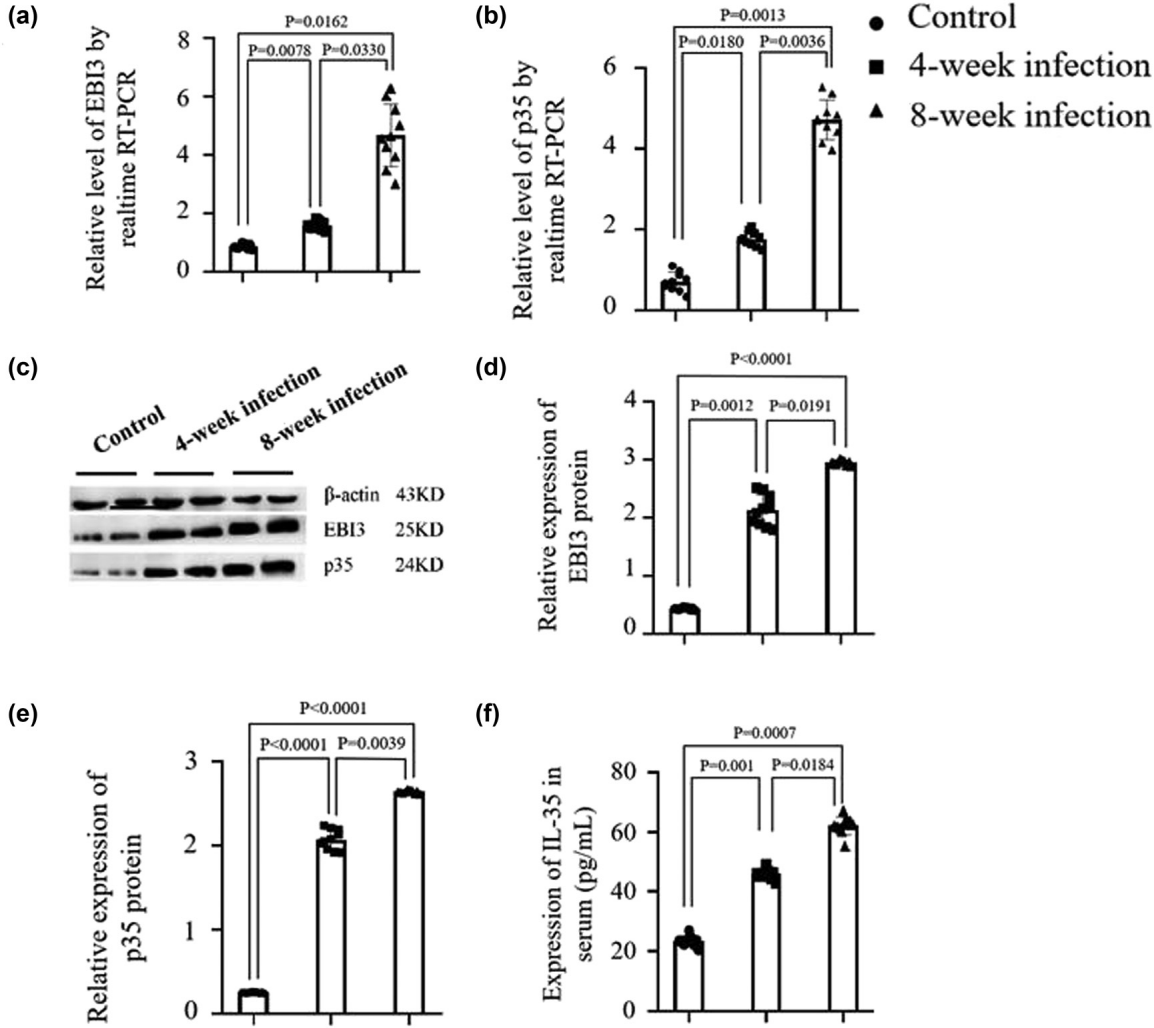
Detection of expression levels of EBI3, p35, and serum IL-35. (a and b) The EBI3 and p35 mRNA expression levels in splenocytes detected by qRT-PCR in the Mtb-infected mice at 4 or 8 weeks post-infection. (c) The expression levels of p35 and EBI3 in the lungs analyzed by western blotting at 4 or 8 weeks post-infection. (d and e) Semiquantification analyses of EBI3 and p35 protein expression levels. (f) The serum IL-35 level tested by ELISA at 4 or 8 weeks post-infection. Each histogram exhibits a set of record for 10 mice, referring to the differences as shown.

To evaluate the level of IL-35 in chronic Mtb-infected mice, we first detected the mRNA expressions of both subunits (p35 and EBI3) of cytokine IL-35. Figure 3a and b indicate the mRNA expression levels of EBI3 and p35 in the spleen. It was found that both EBI3 and p35 expression levels in the spleen of mice were higher at 8 weeks post-infection than those at 4 weeks post-infection, which was significantly increased in EBI3 expression (*p <* 0.00330) and in p35 expression (*p <* 0.00360) compared with that in the normal mice. Figure 3c exhibits the protein expression levels of p35 and EBI3 in the lung tissues, and it was analyzed by western blotting. The statistical analysis of EBI3 protein expression was significantly increased in the 8 weeks Mtb-infected mice in comparison with the 4 weeks Mtb-infected mice (*p <* 0.0191) or in the normal mice (*p <* 0.0001) as is shown in Figure 3d. Figure 3e shows the statistical analysis of P35 protein expression level in the 8 week Mtb-infected mice versus the 4 week Mtb-infected mice (*p <* 0.00039) or versus the normal mice (*p <* 0.0001). The result of subsequent serum IL-35 detection in Figure 3f was similar to that of western blotting. The mouse serum samples were received from the Mtb-infected mice at 8 week post-injection. The tested results indicated the IL-35 level was higher in mice at 8 week post-injection than that at 4 week post-injection or in the serum samples from the mice without Mtb infection, which was statistically significant compared with the 4 week Mtb-infected mice (*p* < 0.0184) and without any infected mice (*p* < 0.0007). The data suggested that the chronic Mtb infection in mice led to increasing both p35 and EBI3 expression levels and serum IL-35 levels.

### 3.4 iTr35 cells/CD4^+^ Foxp3^−^T cell proportion in chronic Mtb-infected mice

**Figure 4:**
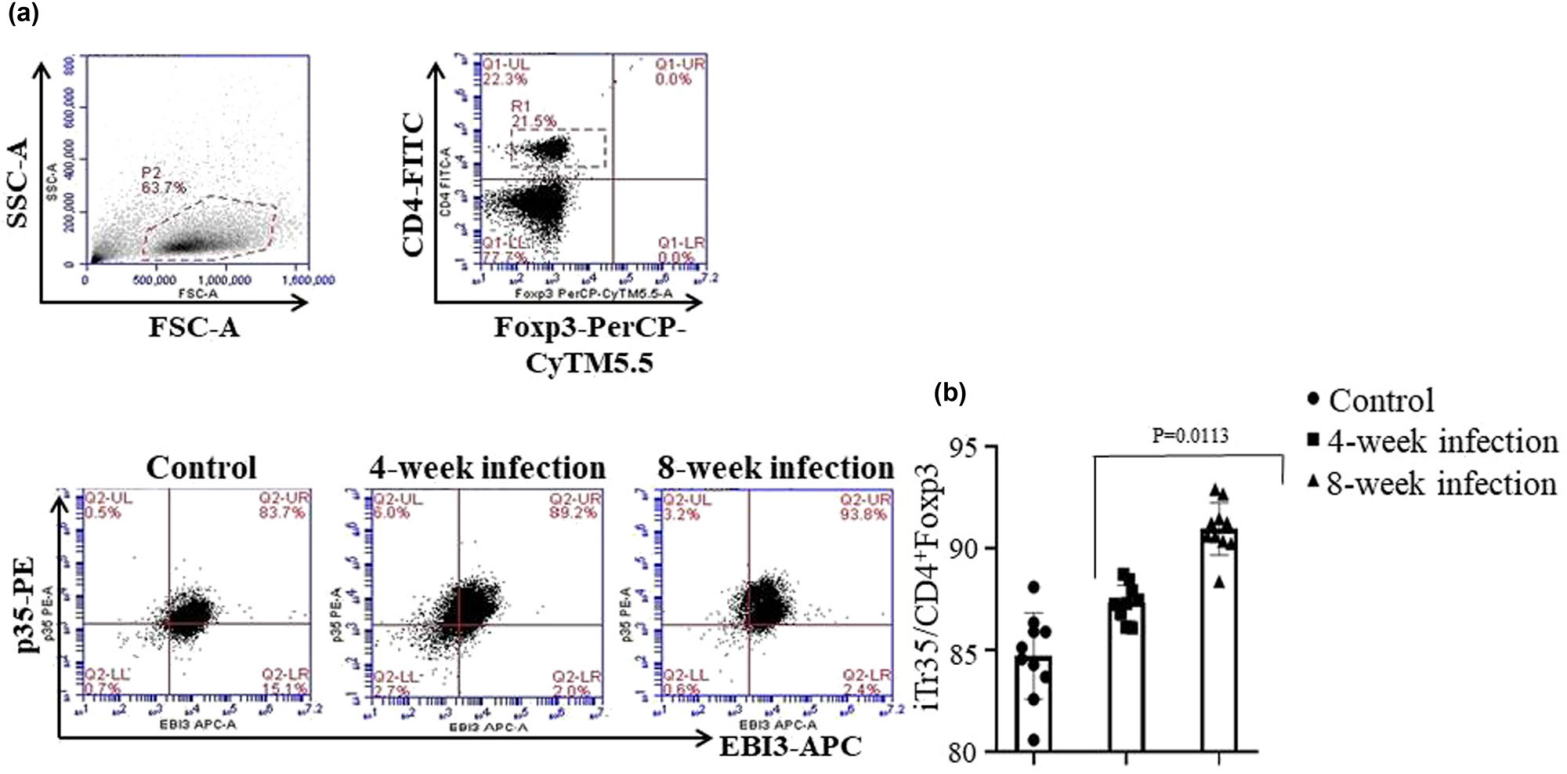
FCM analysis of iTr35 cell subset. (a) The figure represents the iTr35 cell subset in the spleen cells of mice via tail vein injection of attenuated Mtb strain H37Ra at 4 or 8 weeks post-infection. (b) The statistical analysis of total numbers of the iTr35 cell subset in the different group mice analyzed by FCM. The data represent mean ± SD (*n* = 10), referring to the differences as shown.

Next, we investigated the iTr35 cell subset/CD4^+^ Foxp3^−^ T-cell proportion in chronic Mtb-infected mice. Figure 4a shows the results of the iTr35 cell subset analyzed by FCM. It was found that the iTr35 cell subset number in the spleen of Mtb-infected mice at 8 week post-injection was the highest among the three group mice, and the difference was statistically significant between the 8 week infected mice and the control mice (*p <* 0.0113), but there was no statistically significant difference between the 8 week and the 4 week Mtb-infected mice or between the 4 week Mtb-infected mice and the control mice (*p* > 0.05), as shown in Figure 4b. These results indicated that the iTr35 cell subset was increased in the Mtb-infected mice at 8 week post-infection.

## 4 Discussion

TB caused by Mtb is a highly pervasive infectious disease that remains a public health problem worldwide. Owing to an effective vaccine having not been completely developed, researchers have focused on the investigation into the mechanisms of host–pathogen interactions and immune evasion mediated by Mtb chronic infection [25,26]. Cell immune-mediated protection against Mtb can act as a biological tag of developing bacterial infection[27], of which Tregs and regulatory B cells (Bregs) have critical roles as a negative regulator of immunity, mainly due to the fact that they secret a high level of IL-10, transforming growth factor β (TGF-β), and IL-35. From immune evasion mechanisms, the induction of immunosuppression by Tregs and Bregs through IL-10, TGF-β, and IL-35 generates the most effective roles, which optimize the conditions for the survival of pathogens [27,28]. This mechanism is also used by widespread bacteria and viruses that are capable of chronically persisting in the human body, such as Mtb, hepatitis viruses, human immunodeficiency virus, and others [28,29]. However, limited studies are available on the functions of IL-35 and IL-35-inducible Treg (iTR35) cells on chronic infectious TB disease.

For this reason, we first established the chronic infectious mouse model via tail vein injection of attenuated Mtb strain H37Ra. To study and evaluate against tuberculous agents, investigators usually use the Mtb attenuated strain H37Ra, which is like to Mtb strain H37Rv, but the virulence of Mtb strain H37Ra is weaker than Mtb strain H37Rv due to mutation in protein expression or structure, which may have an impact on the virulence property of Mtb H37Ra. However, Mtb H37Ra has a superiority that means the experimental costs is low and the administrative barriers are less such as a biosafety Level III environmental requirement [30,31].

Sequentially, we used the mouse model to observe the change of the IL-35 and IiTR35 cell subset. From the animal experiment results presented in this study, we found that the Mtb strain H37Ra grew well in C57BL/6 mice 4 weeks after injection with 1 × 10^5^ CFU by tail vein, which reflected in decreasing BMI but the spleen coefficient being significantly lowered, the mice showed lung histopathological changes. We found that the IL-35’s subunits p35 and EBI3 expressions, serum IL-35 level as well as IL-35-producing iTr35 cell subset were concurrently increased in the Mtb-infected mouse spleen at 4 or 8 weeks post-infection, especially in 8 week infected mice. It was reported that IL-35 comprises an IL-12p35 subunit encoded by *IL12a* gene, and a β-chain subunit encoded by EBI3 (*IL27b*) connected through a disulfide bond [8], while Treg and Breg cells were the main source of IL-35 that contributes to expansion and suppression activities of these cells, and suppresses Th1 and Th17 proliferation by inducing cell cycle arrest at G1 phase [32,33]. We guess that the change of IL-35 and iTr35 cell subset is closely associated with immune suppression, which may result in aggravating the bacterial load and lung lesions in chronic Mtb-infected mice at 8 week post-infection. This is due to immune suppression effects on T-cell-mediated immune response. Accordingly, avoiding or blocking the immune suppression role mediated by IL-35 and iTr35 cells is an important strategy for evaluating host–Mtb interactions and the efficacy of antibiotics targeting Mtb.

In spite of the above-mentioned findings are promising, the limitations of the present study are that the Mtb infection is how to enhance the IL-35 level and iTr35 cell subset, as well as the analyses of TGF-β, IL-10 levels, other cytokines (e.g. IFN-γ) and immune cell subsets were lacked. Further study that focuses on the mechanism of immune suppression in chronic Mtb-infected mice needs to be conducted.

## 5 Conclusion

In conclusion, the results from the study demonstrate that the established chronic infected mouse model via tail vein injection of attenuated Mtb strain H37Ra at 8-week post-infection caused elevated serum IL-35 level and iTr35 cell subset, accompanied by the increase of bacterial load and lung lesions. Our approach here also supports evidence that the IL-35 level and iTr35 cell subset may be associated with the immune suppression in Mtb-infected mice.
